# Biochemical Mechanisms for Geographical Adaptations to Novel Toxin Exposures in Butterflyfish

**DOI:** 10.1371/journal.pone.0154208

**Published:** 2016-05-03

**Authors:** Aileen Maldonado, Ramon Lavado, Sean Knuston, Marc Slattery, Sridevi Ankisetty, Jared V. Goldstone, Kayo Watanabe, Eunha Hoh, Rama S. Gadepalli, John M. Rimoldi, Gary K. Ostrander, Daniel Schlenk

**Affiliations:** 1 Department of Environmental Science, University of California Riverside, Riverside, California, United States of America; 2 University of Hawaii at Manoa, Honolulu, Hawaii, United States of America; 3 Department of BioMolecular Sciences and the National Center for Natural Products Research, University of Mississippi, University, Mississippi, United States of America; 4 Woods Hole Oceanographic Institute, Woods Hole, Massachusetts, United States of America; 5 Graduate School of Public Health, San Diego State University, San Diego, California, United States of America; University of Siena, ITALY

## Abstract

Some species of butterflyfish have had preyed upon corals for millions of years, yet the mechanism of butterflyfish specialized coral feeding strategy remains poorly understood. Certain butterflyfish have the ability to feed on allelochemically rich soft corals, e.g. *Sinularia maxima*. Cytochrome P450 (CYP) is the predominant enzyme system responsible for the detoxification of dietary allelochemicals. CYP2-like and CYP3A-like content have been associated with butterflyfish that preferentially consumes allelochemically rich soft corals. To investigate the role of butterflyfish CYP2 and CYP3A enzymes in dietary preference, we conducted oral feeding experiments using homogenates of *S*. *maxima* and a toxin isolated from the coral in four species of butterflyfish with different feeding strategies. After oral exposure to the *S*. *maxima* toxin 5-episinulaptolide (5ESL), which is not normally encountered in the Hawaiian butterflyfish diet, an endemic specialist, *Chaetodon multicinctus* experienced 100% mortality compared to a generalist, *Chaetodon auriga*, which had significantly more (3–6 fold higher) CYP3A-like basal content and catalytic activity. The specialist, *Chaetodon unimaculatus*, which preferentially feed on *S*. *maxima* in Guam, but not in Hawaii, had 100% survival, a significant induction of 8–12 fold CYP3A-like content, and an increased ability (2-fold) to metabolize 5ESL over other species. Computer modeling data of CYP3A4 with 5ESL were consistent with microsomal transformation of 5ESL to a C15-16 epoxide from livers of *C*. *unimaculatus*. Epoxide formation correlated with CYP3A-like content, catalytic activity, induction, and NADPH-dependent metabolism of 5ESL. These results suggest a potentially important role for the CYP3A family in butterflyfish-coral diet selection through allelochemical detoxification.

## Introduction

Charles Darwin, in *the Origin of Species*, was the first to emphasize the importance of interspecific interactions in driving the diversification of life. In spite of the long-recognized importance of such interactions, little is known about the diverse system that governs interspecific interactions. The pairwise reciprocal coevolutionary scenario can predict the development of a chemical “arms race”, in which reciprocity between chemical defenses and counter-adaptive detoxification takes place. In marine systems, however, knowledge of genes conferring consumer tolerance to dietary allelochemicals is in its infancy, when compared to that of terrestrial systems [1–3]. Butterflyfish-coral interactions are an ideal model to study consumer tolerance in marine ecosystems because they share many of the same characteristics as plant-insect interactions. Plants and corals are sessile creatures producing allelochemicals to prevent predation [4,5]. Just as insects have preyed upon plant hosts for 400 million years, butterflyfish have likely preyed upon corals over the past 50 million years [6,7]. Coral consumption (corallivory) is a unique adaptation as only 128 fish species eat corals, out of the 5000 or more fish species recorded from coral reefs, and 61% belong to a single-family, the butterflyfish (*f*. *Chaetodontidae*) [6,8]. Despite being one of the most intensively studied families of reef fishes, the evolution of coral feeding remains poorly understood [9].

Among corallivorous butterflyfish, sympatric species often exhibit highly contrasting levels of dietary specialization (S1 Table) [10]. Specialist species tend to have a narrow dietary range, while generalist species are able to thrive on a widely varied diet [11]. Generalists, such as *Chaetodon auriga*, consume species from polychaete worms to hard corals [12], are hypothesized to have liver enzymes that can act on a broad range of substrates to facilitate the biotransformation of a wide variety of toxins. In contrast, generalists consume small amounts of a variety of toxins that are processed through a diverse set of detoxification pathways without overloading any one pathway. This hypothesis has become firmly entrenched in the ecological literature to the extent that it is accepted as the predominant factor regulating the foraging ecology of generalist herbivores e.g. [13]. However, there have been relatively few empirical tests to confirm this hypothesis [13–15]. Specialists, such as *Chaetodon multicinctus*, consume three species of corals [16], limiting their range of available food and potentially increasing their capacity to detoxify specific toxins in high concentrations.

Specialists are thought to have evolved novel liver enzymes with greater specificity [17,18,19–22]. For example *Chaetodon capistratus* preferentially feeds on allelochemically-rich gorgonian corals, while *C*. *striatus* and *C*. *ocellatus* do not feed on gorgonians. *C*. *capistratus* possessed 2- to 3-fold more total CYP and significantly more CYP2B-like and CYP3A4-like proteins when compared to *C*. *striatus* and *C*. *ocellatus* [18]. These results suggest that CYP2 and CYP3 may be involved in allelochemical biotransformation conferring potential feeding advantages [18]. Similarly, phylogenetic evaluations of hepatic CYP1A in Australian butterflyfish indicated grouping according to feeding strategies [1]. Although chemical arms race dogma would suggest consistent benefit to animals with elevated detoxification enzymes, some studies have shown that organisms that detoxify toxins associated with their unique diet may be susceptible to novel toxins. For example, when the woodrats *Neotoma stephensi* (specialists) and *Neotoma albigula* (generalists) were exposed to a novel dietary toxin from *Larrea tridentate*, fitness metrics of the specialists were more negatively impacted than the generalists [14].

Pre-exposure to allelochemicals has been shown to induce biochemical defenses in insects, and specialists have also been shown to have unique CYPs that allow detoxification [19–22]. However, no studies have directly investigated how butterflyfish specialists survive on diets containing novel coral toxins compared to that of generalists fed the same coral toxin. This study investigated the biochemical ability of butterflyfish with different feeding strategies to consume a novel Alcyoniidae soft coral (*Sinularia maxima*) prey, which has been shown to deter feeding in several species of fish. The following questions were asked: (i) whether *S*. *maxima* or the isolated allelochemical from *S*. *maxima* 5-episinuleptolide (5ESL) can deter feeding in butterflyfish with different feeding strategies, (ii) whether *S*. *maxima* or 5ESL can induce CYP in butterflyfish with different feeding strategies, and (iii) whether butterflyfish liver microsomes could biotransform 5ESL into metabolites predicted from CYP(s) that were uniquely induced by 5ESL or *S*. *maxima* extracts.

## Methods

### Compliance with Ethical Standards

The author and co-authors of this paper have acted ethically in conducting the described research, having undertaken careful analysis of data and the submitted manuscript to avoid errors. Animals in this study were treated humanely, with protocols overseen and approved under the University of Hawaii at Manoa, Institutional Animal Care & Use Committee (IACUC), protocol # 13–1701.

### Chemicals

Analytical grade methanol, ethanol and acetonitrile were purchased from Fisher (Pittsburg, PA). Glycerol, Tris(hydroxymethyl)aminomethane and potassium chloride were also purchased from Fisher (Pittsburg, PA). ^14^C-Testosterone (150 μCi/μmol; 97.6% purity) was purchased from Perkin-Elmer (Waltham, MA). MS222, EDTA, gelatin and NAPDH were purchased from Sigma-Aldrich (St. Louis, MO). Tween was purchased from EMD Millipore (Billerica, MA).

### Chemical Synthesis

5,8-epoxy-11-hydroxy-18-nor-3,6-dioxo-12-cembradien-15-methyloxirane-20,10-olide (5ESLO) was synthesized directly from 5-episinuleptilide (5ESL). Sodium bicarbonate was added to a solution of 5ESL (10 mg; 0.003 mmol) in 2 mL of CH_2_Cl_2_, 3.0 mg (0.032 mmol) and the mixture was cooled to 0°C. A solution of *meta*-chloroperbenzoic acid (7.5 mg; 0.032 mmol; 70%) in 1.0 mL of CH_2_Cl_2_ was added slowly over a period of 5 minutes. The resulting slurry was stirred for 30 minutes at 0°C, and then stirred at ambient temperature for 3 hrs, as the progress of the reaction was monitored by TLC (5% CH_3_OH/ 95% CH_2_Cl_2_; ninhydrin staining) and LC-MS (MNa^+^ = 387). The reaction mixture was diluted with an additional 5.0 mL of CH_2_Cl_2_ and was washed twice with saturated sodium carbonate solution (10 mL x2). The organic layer was washed twice with saturated Na_2_S_2_O_3_ solution, dried over anhydrous sodium sulfate, filtered, and concentrated to give 11 mg of crude epoxide product. Purification was accomplished using silica gel and a mobile phase consisting of 5% CH_3_OH:95% CH_2_Cl_2_. Fraction 1 contained unreacted starting material (2.0 mg) and fraction 2 (5.0 mg) corresponded to the epoxide product. ^1^H NMR (400 MHz, CDCl_3_): Δ 6.64 (dd, J = 13.1, 5.6 Hz, 1H), 4.69 (s, 1H), 4.63 (d, J = 7.9 Hz, 1H), 3.89 (d, J = 10.1 Hz, 1H), 3.45 (d, J = 6.8 Hz, 1H), 3.26 (d, J = 6.6 Hz, 1H), 2.49 (dt, J = 17.3, 8.7 Hz, 1H), 2.38 (s, 1H), 2.20 (s, 2H), 2.04 (d, J = 12.7 Hz, 1H), 1.91 (d, J = 3.2 Hz, 1H), 1.51 (s, 1H), 1.39 (s, 1H), 1.25–1.13 (m, 7H), 0.90–0.72 (m, 3H). LC-MS (ESI+) *m/z* (MNa^+^) 387.3.

### Animal Collections

Butterflyfish *C*. *auriga* (generalist), *C*. *unimaculatus* (*Montipora spp*. hard coral specialist in Hawaii and *S*. *maxima* soft coral specialist in Guam), *C*. *multicinctus* (*Porites spp* hard coral. specialist and Hawaiian endemic) and *C*. *kleinii* (facultative coral and plankton feeder) were chosen for this experiment because of their diverse feeding preferences and abundance in Hawaii. Butterflyfish were collected from Kaneohe Bay (*C*. *auriga* and *C*. *unimaculatus*) and Yokahama Bay (*C*. *multicinctus* and *C*. *kleinii*) reef systems, surrounding the island of Oahu during June 2012 and 2013. Both locations required no specific permissions, as it is legal in the state of Hawaii to collect fish in Kaneohe Bay and Yokahama Bay. Butterflyfish species collected in this experiment were not endangered or protected. Professional fish collectors caught butterflyfish with nets. *C*. *multicinctus* (11 ±3 g; 8 ± 0.2 mm), *C*. *kleinii* (23 ± 7 g; 9 ± 1 mm), *C*. *auriga* (43 ± 8 g; 10 ± 1 mm) and *C*. *unimaculatus* (10.4 ±6 g; 7.2 ±1.7 mm) were acclimated for two weeks before treatments. Fish were juveniles and sexually immature by visual determination of the gonads after dissection. Fish were held at the Waikiki Aquarium in a large (15 ft diameter and 3 ft height) flow-through tank, with floating cages for separation of doses. *S*. *maxima* and *S*. *polydactyla* were collected from Piti Bomb holes, Guam and identified by M. Slattery [23].

### 5-Episinuleptolide Isolation

5-ESL was isolated in the same manner as Kamel et al. [24]. In summary, *S*. *maxima* was initially frozen on dry ice, thoroughly extracted with methanol: dichloromethane (1:1), concentrated under reduced pressure, and subjected to silica gel vacuum liquid chromatography. The column was eluted with hexane, hexane–ethyl acetate, ethyl acetate–methanol to methanol to yield 11 fractions, which were concentrated under reduced pressure. 5-ESL was eluted with 80% ethyl acetate:hexane, recrysallized and washed successively with chloroform and methanol.

### Oral Treatments

To examine the effect of *S*. *maxima* tissue homogenate and 5ESL on survival and hepatic CYP expression, 4–6 fish of each species and each dose were gavaged with either high doses (250mg/kg) or low doses (50mg/kg) of *S*. *maxima* tissue homogenate (Summer 2010), or high dose (3.0mg/kg), or low dose (1.0mg/kg) of 5ESL (Summer 2011). 1M Tris-buffer (pH 8.2) served as a negative control. Doses of the compound which is found in *Sinularia* were based on average daily consumption rates of other prey items by *C*. *multicinctus*, *C*. *auriga*, *C*. *unimaculatus* and *C*. *kleinii* e.g. [12,16]. Fish were anesthetized with waterborne MS-222 (0.1 g/L) for 10 min during gavage to reduce stress. Treatments occurred with gavage quickly and efficiently to reduce stress on days 1, 3 and 5. Prior and during treatments fish were monitored bidaily for survival, health and parasites. Fish were euthanized with MS-222 during the experiment if they were immobile or severely floundering. At the end of the treatment period (day 7) fish were given an anesthetic waterborne overdose of MS-222 (10 g/L). After approximately 10–20 minutes of fish gills not moving, fish were removed and dissected. There were no unexpected deaths during this experiment. Livers were removed, frozen and maintained in -80°C until microsomes were prepared for immunoblot and catalytic activity analyses. All experimental manipulations were reviewed and approved by IACUC, protocol # 13–1701.

### Preparation of Microsomal Fractions

Microsomal fractions were prepared as described in Maldonado et al. [25]. Proteins were measured by the Coomassie Blue method using a commercial kit (Pierce Inc., Rockford, IL) with bovine serum albumin as a standard.

### Western Immunoblot

CYP3A-like and CYP2-like protein levels were determined by Western blot as described in Maldonado et al. [25]. The membrane was probed with either 1:500 dilution (v/v) of primary rabbit anti-rainbow trout polyclonal CYP2K1 and/or CYP2M1 or 1:1000 dilution (v/v) of primary rabbit anti-rainbow trout polyclonal CYP3A27 antibodies provided by Dr. Malin Celander University of Gothenburg and Dr. Don Buhler, Oregon State University. Imager ChemiDoc XRS+ Imaging System (BioRad) image analyzer. The data were presented in optical density units per mg protein.

### ^14^C Testosterone Hydroxylase Activity

^14^C Testosterone hydroxylase activity was measured as described by Maldonado et al. [25]. The metabolites were quantified by integrating the area under the radioactive peaks (recovery 98.9%-99.7%); the detection limit was 0.2 pmol/min/mg protein.

### 5-Episinuleptolide Metabolism and Metabolite Identification

Incubations contained 100 μg of hepatic microsomal protein with 5ESL from 1 to 30 μM and included 7.5 μM NADPH in a final volume of 0.204 mL of 50 mM Tris–HCl pH 7.4. Samples were incubated for 45 min at 30°C. Incubation reactions were stopped with 100 μl of acetonitrile, followed by centrifugation at 10,000 g for 10 min. Then 1 μM of the internal standard caffeine was added, and finally 40 μl of supernatant was injected onto a reverse-phase HPLC column. HPLC analyses were performed on an SCL-10AVP Shimadzu HPLC system equipped with a 250Å~4.6 mm Atlantis C18 (5 μm) reverse-phase column (Waters, Milford, MA). Separation of 5ESL metabolites was attained using mobile phase systems of (A) 95% water and 5% acetonitrile and (B) 100% acetonitrile, at a rate of 1 ml/min. The gradient started with 100% A to 50% B in 40–45 min. Chromatographic peaks were monitored with an SPD-10A VP SHIMADZU UV-VS detector (SHIMADZU Co., Carlsbad, CA) at wavelength 249 nm. 5ESL 15,16 oxide and the internal standard were quantified by integrating the area under the peaks using a 6 point standard curve, with a limit of detection of 5 pmol/min/mg protein.

*C*. *unimaculatus* samples were selected for further metabolite characterization by LCMS/MS in positive electrospray ionization mode using a Agilent 6460 triple quadrupole mass spectrometer at gas temperature 300°C and capillary voltage of 2500V. A 1 μL aliquot of metabolite was loaded onto an Agilent Poroshell 120 SB- C18 column (2.1mm x 50mm, 2.7 μm particle size, Santa Clara, CA) with a mobile phase flow rate of 0.2 mLmin−1. Samples were eluted with a gradient composed of 5 mM ammonium acetate, water containing 0.1% formic acid (A) and acetonitrile containing 0.1% formic acid (B). The linear gradient transitioned from a 5% solvent B to 50% for the first 15min, to a gradient of 50% B over 0.2min. Then the initial solvent (5% B) was restored over a 1min linear gradient and re-equilibrated. The ion trap was programmed to collect a MS2 spectrum from 100 to 1000 m/z, a fragment voltage of 20V and a cell acceleration voltage of 7V from collision- induced dissociation of the most intense target ion from the appropriate product ion spectrum. MS data was analyzed with Agilent Mass Hunter (Agilent Technologies, Santa Clara, CA).

### Molecular Modeling

Ligands and crystal structures were prepared for docking using AutoDockTools v1.5.4; [26], with the addition of polar hydrogens and the assignment of Gasteiger charges. Ligand structure and charge minimization was performed with semi-empirical methods (PM6 Hamiltonian in Mopac2009) [27]. Gasteiger-Marsili partial charges were used in the final docking runs. High throughput docking was performed using Autodock Vina (v1.1.2)[28]. Flexible ligands were docked into models with rigid protein backbones and rigid side chains. 100 replicate dockings were performed, retaining a broad range of calculated energies (6 kcal/mol). Docking visualization was performed using PyMol (v. 1.5.0.4, Schrödinger LLC, Portland, OR).

### Statistical Analyses

Statistical analyses were conducted using Prism5 v5.0a software. For the feeding deterrence assay, since the control pellets were typically all consumed (i.e., zero pellets rejected), Fisher’s Exact Test was the most appropriate statistical analysis for this dataset. Prior to statistical analysis, all data was analyzed to meet the normality and variance assumptions of parametric tests. For normally distributed data, an initial one-way ANOVA analysis was carried out to evaluate the differences between doses, not between treatments. If a P-value less than 0.05 was observed, it was considered statistically significant. If significance was determined, the Tukey’s multiple range test was performed to determine differences between groups. If data did not meet assumptions of the parametric test, a Kruskal–Wallis test and two-tailed multiple comparisons Dunn's test was used.

## Results

### *S*. *maxima* Oral Exposure

**Fig 1:**
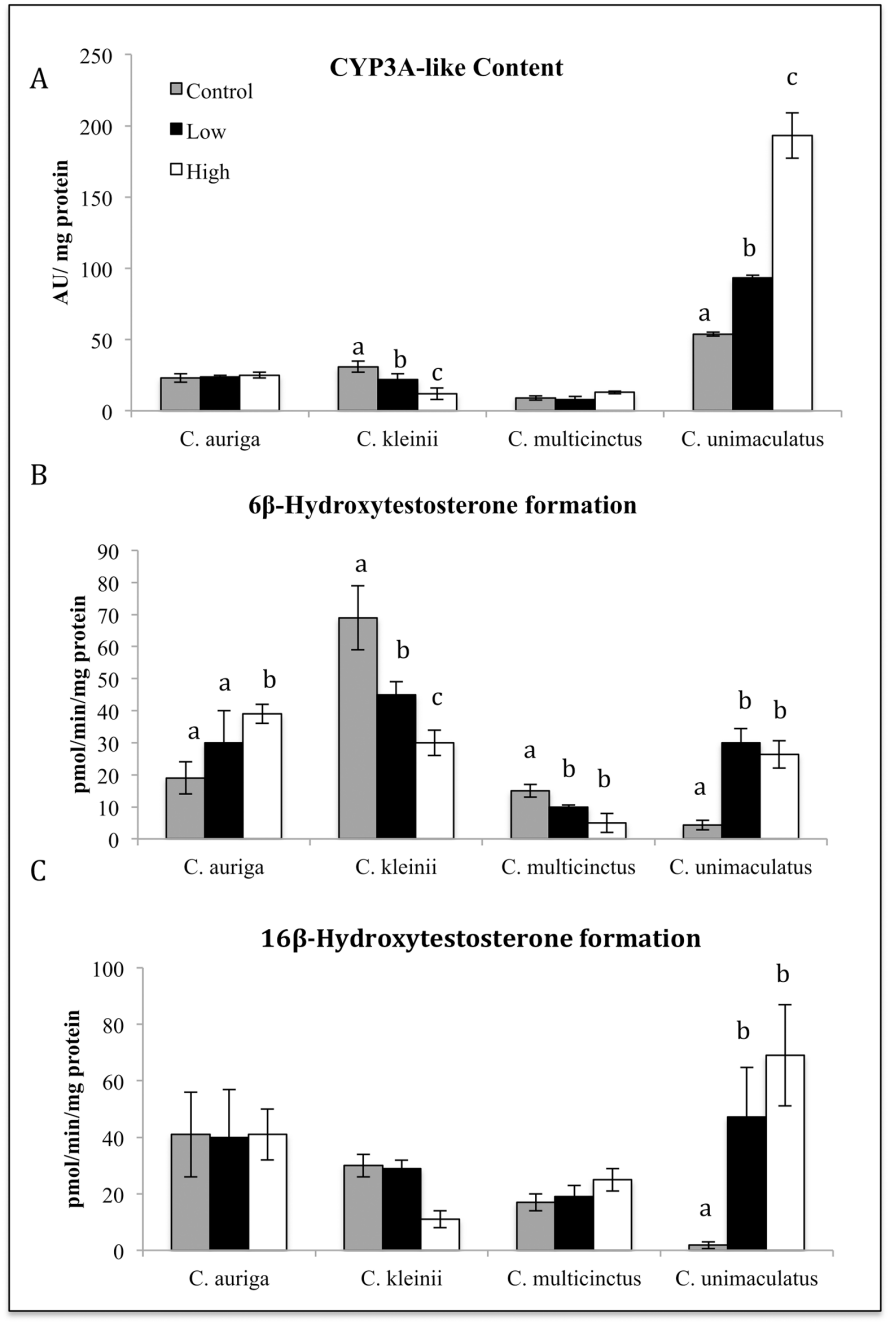
Effects of dietary exposure to *S*.*maxima* tissue homogenate on the hepatic content (A) and catalytic activity (B, C) of and CYP3A in four species of butterflyfish. Different letters indicate significant (P ≤ 0.05) differences between untreated and treated fish (N = 6–8).

**Fig 2:**
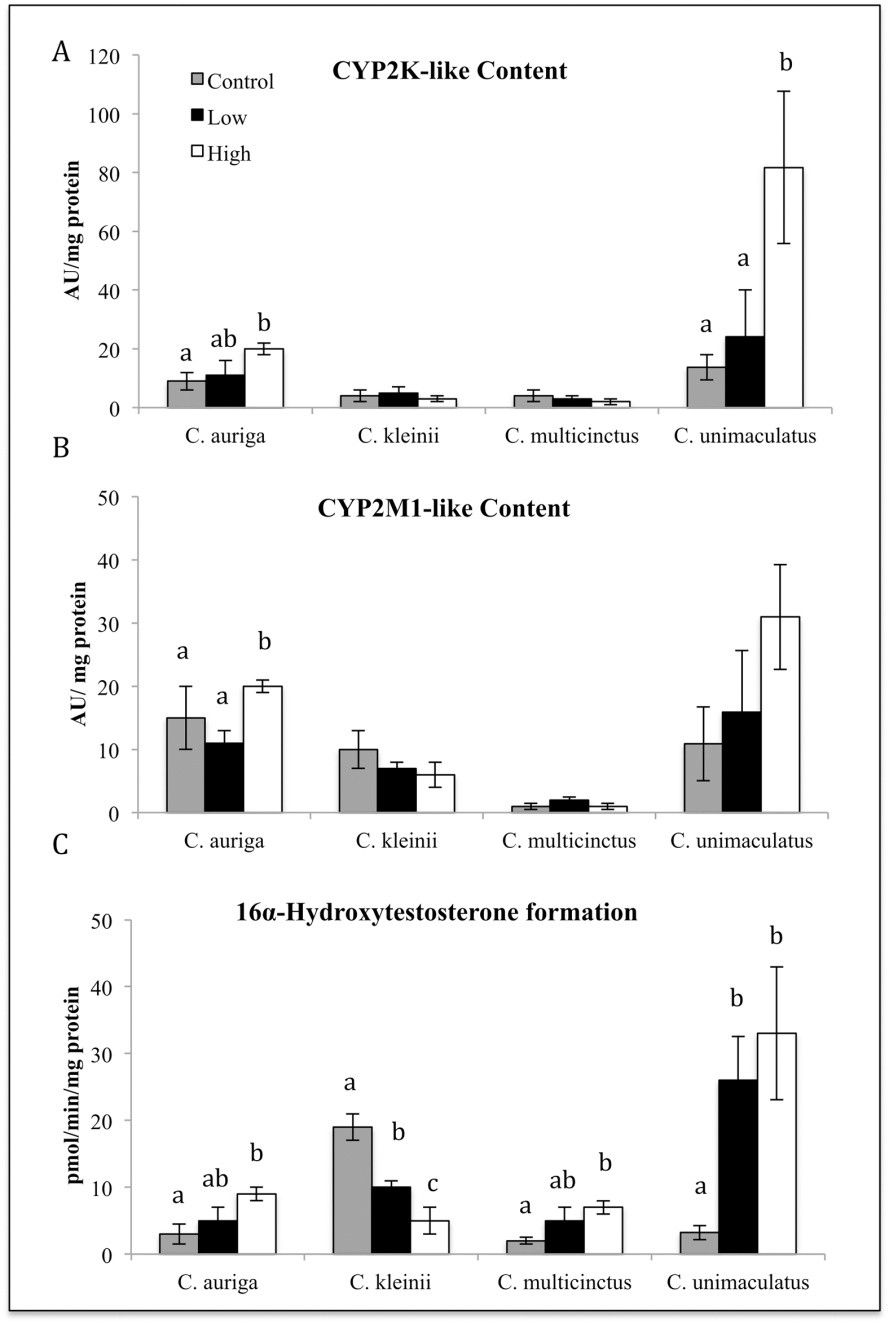
Effects of dietary exposure to *S*.*maxima* tissue homogenate on the hepatic content CYP2K1-like and CYP2M1-like (A, B) and catalytic activity (C) of and CYP2 in four species of butterflyfish. Different letters indicate significant (P ≤ 0.05) differences between untreated and treated fish (N = 6–8).

Survival, hepatic CYP3A-like content, CYPA catalytic activity, CYP2-like content, and CYP2 catalytic activity was measured in 4 species of butterflyfish after oral gavage treatments with of *S*. *maxima*. The four species of butterflyfish included *C*. *auriga* (generalist), *C*. *kleinii* (Facultative coral feeder), *C*. *multicinctus* (specialist), *and C*. *unimaculatus* (specialist). Oral exposure to soft coral *S*. *maxima* tissue homogenate had no significant effect on survival. In *C*. *kleinii*, oral treatment with the *S*. *maxima* tissue homogenate caused a statistically significant dose dependent reduction in CYP3A-like content and catalytic activity (16ß hydroxytestosterone formation) (*P* ≤ 0.05) (Fig 1A and 1C), and a statistically significant dose dependent reduction in CYP2 catalytic activity (16*a*-hydroxytestosterone formation) (*P* ≤ 0.05) (Fig 2C). The formation of 6ß-hydroxytestosterone in *C*. *auriga* was also induced by *S*. *maxima* at the high dose by a factor of 2.3 (Fig 1B). *C*. *auriga’s* CYP2K-like content and 16*a*-hydroxytestosterone formation in *C*. *auiria* were significantly induced by *S*. *maxima* tissue homogenate at the high dose with a 2.33-fold change in content and a 3.2-fold change in catalytic activity (*P* ≤ 0.05) (Fig 2A and 2C). Oral exposure to *S*. *maxima* caused a significant 1.5-fold increase in *C*. *multicinctus* CYP2-like activity (16a-hydroxytestosterone) at the high dose (Fig 2C). *C*. *multicinctus* 6ß-hydroxytestosterone formation (CYP3A activity) was significantly decreased by the high dose 2.5-fold (Fig 1B). Oral treatment of fish with *S*. *maxima* tissue homogenate caused a significant induction in the formation of 6ß, 16ß and 16*a-*hydroxytestosterone (10–20 fold) (Figs 1B,1C and 2C) and CYP3A-like and CYP2-like content (5–11 fold) in *C*. *unimaculatus* (Fig 1A), but caused no significant changes in *C*. *auriga*. In *C*. *unimaculatus*, CYP3A catalytic activity (16ß and 6ß hydroxytestosterone formation) was induced 4-fold in protein and 2-fold in activity (*P* ≤ 0.05) (Fig 2A–2C).

### 5ESL Oral Exposure

**Fig 3:**
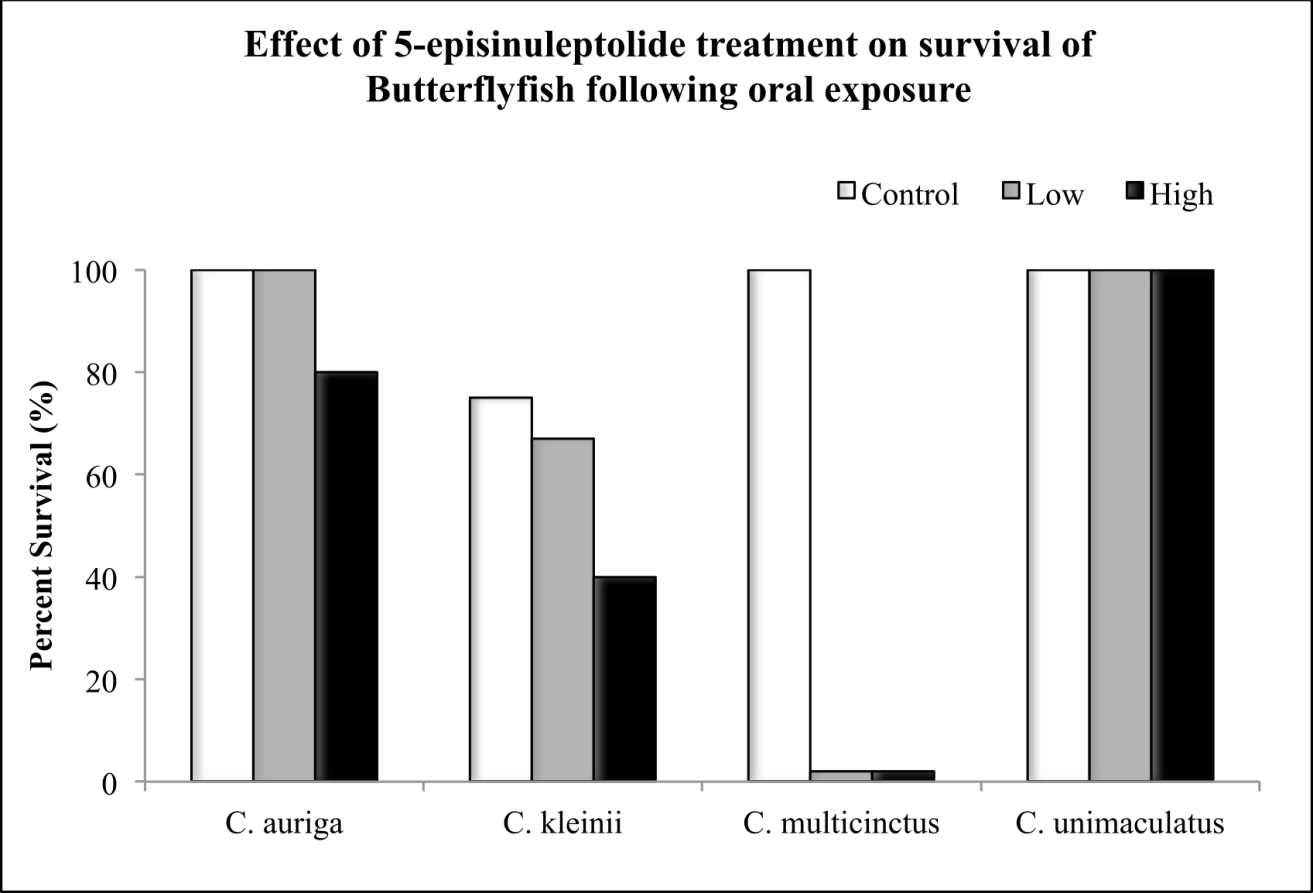
The percentage survival of butterflyfish after a 7 day oral exposure to high dose (3.0mg/kg), or low dose (1.0mg/kg) of 5ESL. Each value represents the total survival of 6–8 individuals.

**Fig 4:**
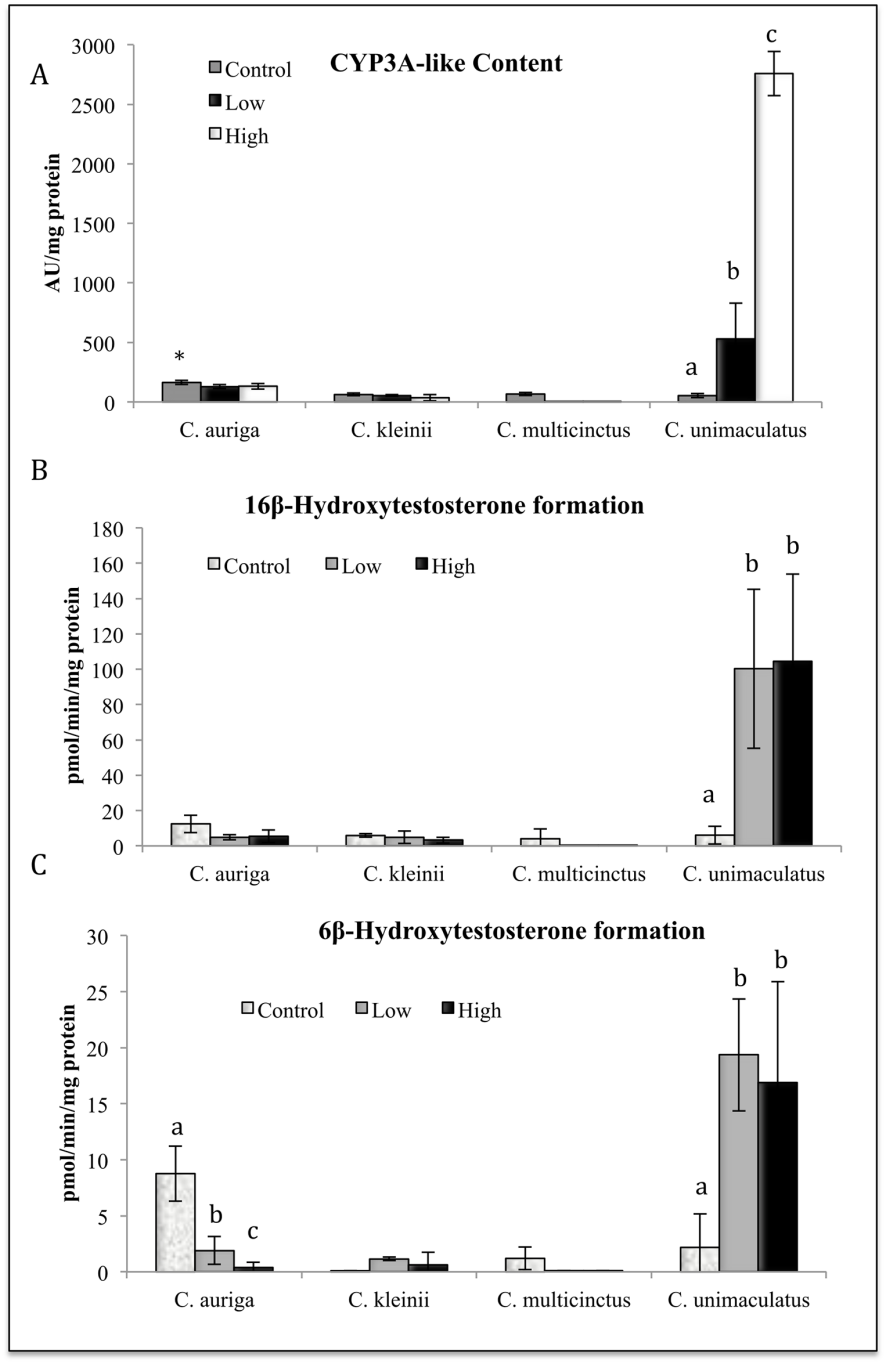
Effects of dietary exposure to 5ESL on the hepatic content (A) and catalytic activity (B, C) of and CYP3A in four species of butterflyfish. Different letters indicate significant (P ≤ 0.05) differences between untreated and treated fish (N = 6–8).

**Fig 5:**
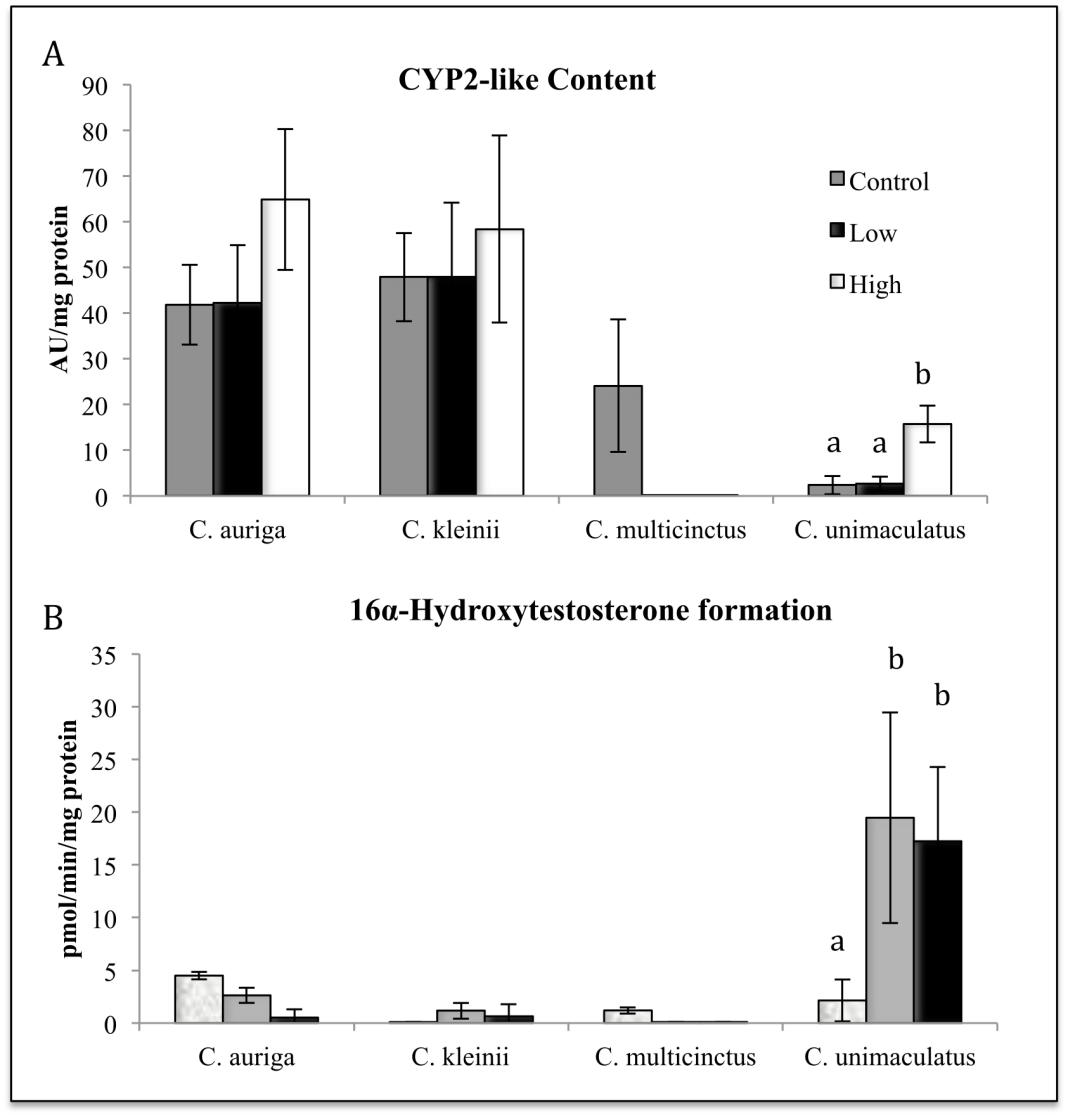
Effects of dietary exposure to 5ESL on the hepatic content of CYP2M1-like protein (A) and catalytic activity (B) of and CYP2 in four species of butterflyfish. Different letters indicate significant (P ≤ 0.05) differences between untreated and treated fish (N = 6–8).

Survival, hepatic CYP3A-like content, CYPA catalytic activity, CYP2-like content, and CYP2 catalytic activity was also measured in the same 4 species of butterflyfish after gavage treatment of 5ESL.During the oral 5ESL treatments, *C*. *multicinctus* experienced 100% mortality in both the high and the low doses, with 100% survival in the control (Fig 3). In contrast, 80% survival was observed in *C*. *auriga*, with 45–60% survival observed for *C*. *kleinii* after treatment (Fig 4). *C*. *unimaculatus* had 100% survival in all treatments (Fig 3). 5ESL caused 1.3-fold induction of CYP3A-like content in *C*. *auriga*, (*P* ≤ 0.05) but a reduction by a factor of 7.2 in CYP3A catalytic activity (6ß hydroxytestosterone) (*P* ≤ 0.05) (Fig 4A and 4C). 5ESL also caused a reduction in CYP2 catalytic activity by 0.5-fold at the high dose (*P* ≤ 0.05) (Fig 5B). In *C*. *kleinii*, no statistically significant changes in content or catalytic activity were observed after exposure to 5ESL. Due to excessive mortality, liver microsomes from only the *C*. *multicinctus* control group were evaluated for CYP activity and expression. Hepatic CYP3A-like content from *C*. *unimaculatus* had the most significant dose dependent induction following 5ESL treatment compared to the other species, with 8–12 fold times larger values observed after treatment (*P* ≤ 0.05) (Fig 4A). The induction of CYP content in *C*. *unimaculatus* correlated with catalytic activity of CYP3A, increasing 20–25 fold (R^2^ = 0.66728) (Fig 4B and 4C).

### 5ESL Docking Experiment

**Fig 6:**
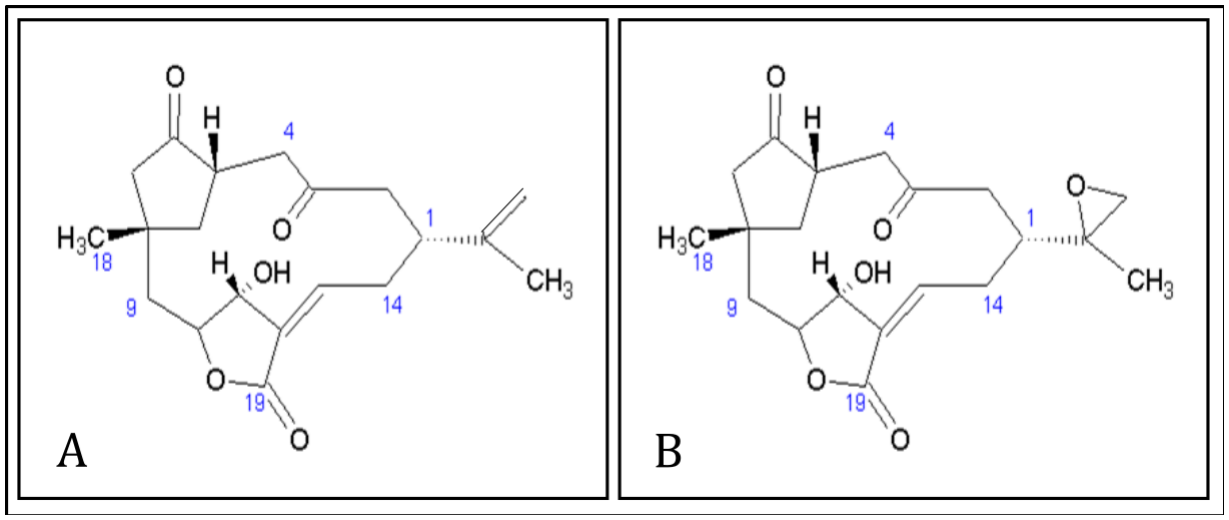
Structures of 5ESL (A) of *S*. *maxima* and 5ESL 15,16 epoxide (5ESLO) (B).

5ESL was docked into a crystal structure of CYP3A4, (PDB:3NXU) with the ligand removed, to assess the ability of CYP3A4 to metabolize 5ESL. Several low energy (high affinity) positions were found well within the oxidizable distance to a computed Fe-O position. In general, two main docking areas were found—one with an oxidizable carbon within 2–3 A of the Fe-bound oxygen, and the other at about a 7–8 A distant. Both could be occupied at the same time, as the Hill coefficient for CYP3A4 is usually 2 (that is, the reaction rate of CYP3A4 with substrate is dependent on the square of the substrate concentration [S] in Michaelis-Menten type kinetics, implying double occupancy of the active site). There were four different carbons (C16, C12, C10, C19) that could be oxidized depending on preferred ligand orientation in the active site, one of which was an epoxide formed on the C15-16 (Fig 6B).

### 5ESL Microsomal Incubations

To investigate the metabolism of 5ESL, liver microsomes of *C*. *auriga*, *C*. *unimaculatus*, *C*. *kleinii*, *C*. *multicinctus* and human CYP3A4 supersomes were incubated with 5ESL and the metabolites were identified. Microsomal in vitro incubations with 5ESL (Fig 6A) showed NADPH-catalyzed clearance in *C*. *auriga*, *C*. *unimaculatus*, *C*. *multicinctus* and human CYP3A4 supersomes (Table 1). Enzyme saturation as determined by Michaels-Menten kinetics occurred at 11 μM. An increased trend for biotransformation of 5ESL with NADPH was observed in the following species: in increasing order—*C*. *multicinctus* < *C*. *kleinii* < *C*. *auriga* < *C*. *unimaculatus;* mimicking the survival trend. Human CYP3A4 completely metabolized 5ESL when incubated with NADPH after 45 min (Table 1). There were three significant peaks at retention times of 2, 3 and 4.5 minutes in the HPLC chromatogram. We further analyzed the peak at retention time 3 min using LCMS/MS and compared the chromatogram to the synthesized 5ESL 15–16 epoxide (5ESLO) (Fig 6B) based on docking data. A 365 m/z molecular ion was detected with the metabolite at 3 min and a purified standard of 5ESLO. Loss of 114 and 247 m/z was observed in both compounds from the corresponding pseudomolecular ions. The structures of the remaining metabolites remain unknown.

**Table 1:** NADPH-dependent clearance and transformation of 5ESL to 5ESLO in liver microsomes from four species of butterflyfish and recombinant human CYP3A4. Different letters indicate significant (P ≤ 0.05) differences between species* indicated signifcnat differences between control and pretreated *C*. *unimaculatus* (P ≤ 0.05) (N = 6–8).

Species	5ESL Clearance	5ESLO formation
	(pmol min^-1^ mg^-1^ protein)	(pmol min^-1^ mg^-1^ protein)
*C*. *auriga*	266.00±51.2	57.39±12.48
	A	A
*C*. *kleinii*	N.D.	17.39± 4.92
		B
*C*. *multicinctus*	13.30±4.22	N.D.
	B	
*C*. *unimaculatus*	490.32±49.3	50.43±15.35*
	C	A
*C*. *unimaculatus*	N.M.	918.75±131.2*
Pre-treated with 5ESL	D	
Human CYP3A4	1125.1±113.4	

Activities were determined using 11 μM 5ESL. Values are represented as the mean ± SD (n = 6–8). N.D. = Not detected; N.M. = Not measured.

### 5ESL Epoxide Formation

After identifying 5ESL15-16 Oxide (5ESLO) as a metabolite, the rates of 5ESLO formation were calculated in liver microsomes of *C*. *auriga*, *C*. *kleinii* and *C*. *unimaculatus*. 5ESLO formation correlated with CYP3A activity, with an R^2^ = 0.862. The highest 5ESLO formation was seen in microsomes obtained from *C*. *unimaculatus* exposed to the high dose of 5ESL relative to untreated *C*. *unimaculatus*, *C*. *auriga* and *C*. *kleinii* (*P* ≤ 0.05) (Table 1). Formation of 5ESLO was below the limit of detection in microsomes from *C*. *multicinctus*.

## Discussion

Previous studies have found that generalist feeding is deterred by allelochemicals, whereas specialists are either unaffected or attracted to the allelochemicals of their preferred diet [29,30]. The capability of *S*. *maxima* to deter predators under laboratory and field conditions has been well described e.g. [23,31]. However, few studies have looked at the effect of different feeding strategies within the same genus or species on deterrence. Homogenates of *S*.*maxima* and the individual compound, 5ESL have been shown to be deterrent to generalists but not specialists (Slattery et al., submitted). Deterrence in the generalists may indicate possible toxicity by learned avoidance in the wild or distastefulness during exposure [32–35]. In contrast, the lack of deterrence in specialist species of butterflyfish may indicate an evolved adaptation to the toxins in the soft coral *S*. *maxima*. *C*. *unimaculatus* preferentially feed on the soft coral *S*. *maximus* in Guam [32] but not in Hawaii where the coral does not occur [36].

In Hawaii, exposure to homogenates of the soft coral *S*. *maxima* had no significant effect on survival or noteworthy effects on CYP2 or CYP3A-like proteins or activities on all 4 species. In contrast, 5ESL treatment caused significant mortality in *C*. *multicinctus* and *C*. *kleinii*. However, survival in *C*. *auriga* following exposure to 5ESL was unaffected. Since survival correlated with basal CYP3A-like and CYP2-like content and activities, CYP3A and CYP2 of *C*. *auriga* may function as multipurpose detoxification enzymes of dietary chemicals. If the fish is capable of detoxifying a broad range of lipophilic compounds, this species of fish may exploit a larger range of prey as a generalist feeder [13–15,37]. Conversely, *C*. *multicinctus*, with a limited range of available food may have CYP’s with greater specificity [21,22,38]. A potential negative effect of this trait is that the specialists are less energetically and/or mechanistically efficient at eliminating novel toxins that are not associated with their specific prey [35]. Consistent with this hypothesis, *C*. *multicinctus* had the lowest survival of all four species and the lowest CYP2-like and CYP3A-like basal content and activities. An example of specialist herbivores being negatively impacted by novel defense compounds is the woodrat, *Neotoma stephensi* that had reduced locomotor activity when exposed to novel plant toxins from *Larrea tridentata* [14,19].

Since there were multiple CYP2-like isoforms observed in western blots, and catalytic activities associated with CYP2 (16*α*-testosterone hydroxylase) were not correlated with survival, focus was placed upon CYP3A as a potential enzyme for detoxification. 5ESL docking into the crystal structure of CYP3A4 showed several low energy (high affinity) positions where four different carbons could be oxidized, depending on preferred ligand orientation in the active site. CYP3A enzymes are functionally among the most versatile forms of CYPs. Mutation and docking studies have demonstrated that CYP3A proteins have a large substrate-binding pocket in comparison to other members of the CYP superfamily [37]. This large pocket may enable CYP3A enzymes to catalyze biotransformation of the large 5ESL molecule. Further analysis revealed the C15-16 epoxide (5ESLO) (Fig 6B) to be the most likely CYP3A metabolite. The 5ESLO metabolite in microsomal in vitro incubations showed NADPH dependent formation of a C15-16 epoxide confirmed by LCMS with a synthesized standard. In addition, formation of the epoxide correlated with hepatic CYP3A-like content and survival in all species treated with 5ESL. This is consistent with the possibility that epoxide formation of 5ESL by CYP3A is a detoxification pathway.

The corresponding pattern of CYP3A-like content induction and catalytic activity in the liver enzymes of *C*. *unimaculatus* dosed with 5ESL is similar to cases in plant-insect interactions, where black swallowtails (*Papilio polyxenes*) where exposed to the plant alleochemical, xanthotoxin that induced CYP activity in a dose-dependent manner, increasing seven fold [21]. Allelochemicals often act as ligands to induce their own CYP metabolism, [19–22]. Although it is unclear how 5ESL regulates CYP3A, other studies have demonstrated that CYP3A can be induced by allelochemicals that bind the nuclear pregnane X receptor (PXR) [39–41]. Further research is needed to elucidate this mechanism.

*C*. *unimaculatus* had the greatest clearance of 5ESL, the highest induction of CYP3A-like content as well as activity, and the highest relative formation of 5ESLO. *C*. *unimaculatus* preferentially feeds on *S*. *maxima* in Guam and Australia, suggesting the fish has a high tolerance for soft coral toxins regardless of current feeding preferences. Induction of CYP3A after exposure to a dietary item that is geographically removed from Hawaii suggests that *C*. *unimaculatus* may have migrated from Guam (where the fish feeds on the soft coral) to Hawaii. The Biodiversity Feedback model indicates that biodiversity flows eastward from the indo-pacific archipelago to peripheral habitats in Hawaii [42]. The Central Pacific area is characterized by low species departure (10–16% Central Pacific), but high dispersal of lineages into the region, from the Indo-Pacific Archipelago [42]. In terms of global diversity for Chaetodontidae, the Indo-Pacific Archipelago stands out as a significant source of diversity in terms of both origination within the region and the expansion of lineages into adjacent regions [42]. Gene flow of two related coral-feeding butterflyfish was found to be high, across the Pacific Ocean over both recent and historical timeframes, in the specialist, *Chaetodon trifascialis* and more stable in the generalist, *C*. *lunulatus* [35]. It is possible that *C*. *unimaculatus* has gene flow between Hawaii and the rest of the Pacific or historically migrated from Guam eastward to Hawaii. Similar hypotheses have been proposed for butterflyfish from other areas of the Indo-Pacific [43] as well as other fish species [44]. Future phylogenetic studies on *C*. *unimaculatus* should be conducted to support or refute the evolutionary migration from Guam to Hawaii.

*C*. *multicinctus* had the least metabolism of all species tested, and it is endemic to Hawaii, with no evolutionary history with *S*. *maximus*. The distinct levels of allelochemical tolerance may explain coral-feeding butterflyfish dietary specialization, since nutritional value of corals has yet to explain these differences [10]. The risks associated with dietary specialization are thought to be offset by increased nutritional benefits (e.g growth) when feeding on preferred prey over generalist feeding on the same prey [45]. However, a study conducted on coral-feeding butterflyfish found that a more specialized species (*Chaetodon trifascialis*) did not outperform the generalist species (*Chaetodon plebeius*) when both consumed their preferred prey [10]. In the current study, *C*. *unimaculatus* appears to have an innate ability to tolerate toxic dietary allelochemicals allowing them to explore uncompetitive diets of soft corals throughout the Pacific [46]. Losing this advantage may have led *C*. *unimaculatus* to consume hard coral *Monipora spp*. in Hawaii [47]. This may also explain the innate inability of *C*. *multicinctus* to tolerate the soft coral toxin 5ESL, and its preference for hard corals. These differences in dietary choices may be explained by their characteristic ability to tolerate dietary allelochemicals.

In conclusion, the use of a toxicological approach has resulted in the identification of butterflyfish cytochrome P450s, specifically CYP3A, as a potential detoxification enzyme. We provide several lines of evidence that implicate inducible butterflyfish CYP3As in the mechanism of adaptation to soft coral allelochemicals. The corresponding pattern of protein content responsiveness and testosterone hydroxylase activity in the liver microsomes of butterflyfish orally exposed to 5ESL coupled with in vitro incubations, computer modeling data, and the formation of 5ESL epoxide metabolite strongly indicates a role for the Chaetodon CYP3As in mediating the metabolism of dietary norcembranoids. The results of our study also demonstrate that functional versatility of counter-defense genes may facilitate generalist (*C*. *auriga*), whereas functional specialization of *C*. *multicinctus* genes may hinder the ability to detoxify novel toxins. Other genetic and molecular factors may contribute to generalist ability to detoxify novel toxins. Comparative gene sequencing surveys of host use-related genes between pairs of closely related butterflyfish species that diverge in their degree of feeding specialization may resolve the question of whether multiple gene duplication events also aid generalists in coping with their diverse and unpredictable coral defense challenges. This work demonstrates the utility of incorporating molecular approaches to better understand the biochemical novelties that allow marine consumers to detoxify allelochemically defended prey. Evolution of host range has been a central interest in the field of butterflyfish ecology; however, little evidence to support theories proposed to explain macroevolutionary patterns of host use and dietary breadth leave a gap in the science that requires more multidisciplinary involvement [48]. Moreover, identifying the molecular foundations of organismal detoxification has broad implications for understanding the role of species-species interactions on gene function.

## Supporting Information

S1 Table Major dietary habits of coral reef butterflyfish. The four species utilized in the exposure experiments are listed with distribution, diet in those locations, and references.
(DOCX)
