# Supplementary material for: Biochemical Mechanisms for Geographical Adaptations to Novel Toxin Exposures in Butterflyfish
Source: PLoS One. 2016 May 3;11(5):e0154208. doi: 10.1371/journal.pone.0154208 (PMC4854401; doi:10.1371/journal.pone.0154208)
Supplement: S1 Table — The four species utilized in the exposure experiments are listed with distribution, diet in those locations, and references. (DOCX) [file pone.0154208.s001.docx]

**S1 Table.**

| Species | Distribution | Diet | References |
| --- | --- | --- | --- |
| *C. auriga* | Indo-Pacific | Generalist | Pratchett et al., 2005 |
| *C. kleinii* | Indo-Pacific | Facultative corallivore | Pratchett et al., 2005 |
| *C. multicinctus* | Hawaii | Obligate hard corallivore | Tricas, 1989 |
| *C. unimaculatus* | Indo-Pacific | Obligate hard corallivore Hawaii  Obligate soft corallivore Guam | Cox, 1994  Wylie et al., 1989 |
